# Convergent phenotypic evolution of the visual system via different molecular routes: How Neotropical cichlid fishes adapt to novel light environments

**DOI:** 10.1002/evl3.71

**Published:** 2018-07-17

**Authors:** Andreas Härer, Axel Meyer, Julián Torres‐Dowdall

**Affiliations:** ^1^ Zoology and Evolutionary Biology, Department of Biology University of Konstanz Germany; ^2^ Radcliffe Institute for Advanced Study Harvard University Cambridge Massachusetts 02138; ^3^ Zukunftskolleg, University of Konstanz Konstanz Germany

**Keywords:** Color vision, crater lake, cyp27c1, molecular adaptation, Nicaragua, opsins, predictability of evolution, regulatory change

## Abstract

How predictable is evolution? This remains a fundamental but contested issue in evolutionary biology. When independent lineages colonize the same environment, we are presented with a natural experiment that allows us to ask if genetic and ecological differences promote species‐specific evolutionary outcomes or whether species phenotypically evolve in a convergent manner in response to shared selection pressures. If so, are the molecular mechanisms underlying phenotypic convergence the same? In Nicaragua, seven species of cichlid fishes concurrently colonized two novel photic environments. Hence, their visual system represents a compelling model to address these questions, particularly since the adaptive value of phenotypic changes is well‐understood. By analyzing retinal transcriptomes, we found that differential expression of genes responsible for color vision (cone opsins and *cyp27c1*) produced rapid and mostly convergent changes of predicted visual sensitivities. Notably, these changes occurred in the same direction in all species although there were differences in underlying gene expression patterns illustrating nonconvergence at the molecular level. Adaptive phenotypes evolved deterministically, even when species differ substantially in ecology and genetic variation. This provides strong evidence that phenotypic evolution of the visual system occurred in response to similar selective forces of the photic environment.

**Impact Summary**

Almost 30 years ago, the famous paleontologist and evolutionary biologist Stephen J. Gould argued that contingency plays a dominant role in evolution and proposed that if we were to replay the tape of life, the world would look quite different and most likely lack humans. Others, such as Simon Conway Morris, have argued that evolution is deterministic and that human‐like, intelligent beings are unavoidable evolutionary outcomes. This debate remains largely unsolved and while Gould's view might hold true over long time scales, evidence is accumulating that over short‐time scales evolutionary change can actually be deterministic, in particular when natural selection is strong. Natural experiments, in which ecologically differentiated species colonized similar or, ideally, the same novel environment provide valuable insights into the roles of determinism and contingency in phenotypic evolution. In this study, we took advantage of such a natural experiment to ask whether multiple species show similar phenotypic change in their visual system in response to shared selection due to the concurrent colonization of new environments. We focused particularly on the visual system since these environments differ dramatically in their light conditions and because there is a good understanding of the physics and chemistry of vision. This, in turn, enabled us to link molecular changes to phenotypic peculiarities. In our study species, expression changes of genes responsible for color vision caused adaptive and mostly convergent shifts in predicted visual sensitivities. However, the set of differentially expressed genes that caused these shifts varied among lineages. At the time scale observed in our system, phenotypic evolution appears to be deterministic, but we want to emphasize the importance of contingency in the underlying gene expression patterns. Thus, we conclude that both contingency and determinism are important factors in evolution and largely depend on the level of biological organization.

What are the relative contributions of deterministic (natural selection) and stochastic (e.g., random mutations, genetic drift, or environmental fluctuations) factors during evolution? Or, phrased differently, how predictable is evolution? This major question has been addressed at different levels of biological organization since convergent evolution was recognized to be omnipresent in all evolutionary lineages. Stephen J. Gould asked this in his famous thought experiment of “replaying life's tape” (Gould 1989). He concluded that stochastic factors were predominant and would preclude predictability of evolutionary change (Gould 1989). Gould's question remains unsolved and is as vexing and current today as it was almost 30 years ago (Conway Morris 2003; Losos 2017). Although it is merely theoretical, a simplified form could be addressed by assessing predictability of evolutionary change across different temporal and phylogenetic scales (Orgogozo 2015). Specifically, one could ask (i) how predictable phenotypic change is when ecologically differentiated lineages are exposed to similar environmental conditions, (ii) and more precisely, whether there is convergence among such lineages with respect to both direction and magnitude of change, and (iii) if the same molecular mechanisms, that is structural or regulatory changes of the same genes, underlie convergent changes.

Certain aspects of evolution are stochastic, including random mutations, genetic drift, and environmental fluctuations. Yet, natural selection is deterministic and allows predicting evolutionary change when selective agents are known. Repeated evolution of similar phenotypic traits in independent lineages, termed convergence (sensu Arendt and Reznick 2008), provides strong evidence for natural selection. Examples of convergence are the repeated loss of armor plates in threespine sticklebacks (Colosimo et al. 2005), changes in body shape in Anolis lizards (Mahler et al. 2013), threespine sticklebacks (Schluter and McPhail 1992; Rundle et al. 2000) and cichlid fishes (Meyer 1990; Elmer et al. 2010a; Elmer et al. 2014) and life‐history evolution in guppies (Reznick and Endler 1982; Reznick et al. 1996). These cases, in which populations of the same, or closely related species, independently colonized similar environments and predictably and repeatedly diverged from the ancestral state have provided valuable insights into how adaptive evolution proceeds. However, hidden environmental heterogeneity might confine the extent of convergence (Fitzpatrick et al. 2014; Stuart et al. 2017). A compelling, but less frequently applied approach is to study convergent evolution of certain phenotypic traits in lineages that concurrently colonized the same environment (Rosenblum 2006; Rosenblum et al. 2017).

Still, identifying the molecular mechanisms underlying convergent phenotypic changes proves challenging, particularly since the phenotypic effects of molecular changes are rarely understood in nongenetic model systems. Further, adaptive evolution can result from nonsynonymous substitutions in coding regions of genes (Hoekstra and Coyne 2007) or by changes in regulatory regions that modify gene expression patterns (Carroll 2005; Wray 2007), which might differ case by case. Since regulation is more modular in its organization, mutations are less likely to have negative pleiotropic effects (Stern and Orgogozo 2008). Therefore, regulatory regions might harbor more standing genetic variation and selection on this (previously neutral) variation is thought to promote rapid adaptation (Stone and Wray 2001; Innan and Kim 2004; Barrett and Schluter 2008; Leder et al. 2015). This, in turn, predicts that during early stages of divergence, most phenotypic differences among lineages will be produced by regulatory rather than structural changes (Ghalambor et al. 2015; Leder et al. 2015). In the longer term, mutations altering protein structures could occur and get fixed, thus, both structural and regulatory differences are expected during later stages of divergence. To further comprehend these general evolutionary processes, we need to identify the mechanisms producing adaptive phenotypic changes.

The visual system represents a fascinating model for studying convergent adaptive evolution since (i) it is highly variable, (ii) the phenotypic effects of molecular changes are well‐understood (reviewed in Bowmaker 2008), (iii) there is a good understanding of the adaptive value of visual phenotypes under certain environmental conditions, and (iv)–as in the case of the natural experiment in Nicaraguan lakes where multiple species have concurrently colonized novel photic environments–one can test how deterministic evolution is. Among vertebrates, cichlid fishes show a remarkably high visual system diversity (Carleton et al. 2016) and molecular mechanisms facilitating adaptive evolution have been studied extensively (Terai et al. 2006; Hofmann and Carleton 2009; Hofmann et al. 2009; Schulte et al. 2014; Torres‐Dowdall et al. 2015; Hauser et al. 2017; Torres‐Dowdall et al. 2017). Color vision is particularly variable in cichlids and is mediated by visual pigments located in photoreceptor cells (cones) of the retina, which are composed of a light‐absorbing chromophore that is covalently bound to a transmembrane opsin protein (Wald 1968; Yokoyama 2000; Ebrey and Koutalos 2001). Cichlids have two types of cone cells, single and double cones, which are characterized by expression of short (*sws1*, *sws2b*, *sws2a*) and medium to long wavelength sensitive cone opsins (*rh2b*, *rh2a*β, *rh2a*α, *lws*), respectively (Fernald 1981; Carleton and Kocher 2001; Hofmann et al. 2009). Shifts in visual sensitivity are mainly produced by structural changes or differential expression of opsins (Terai et al. 2006; Hofmann et al. 2009; O'Quin et al. 2010; Torres‐Dowdall et al. 2017). Additionally, many aquatic vertebrates change visual sensitivity by differential use of vitamin A1 and A2 derived chromophores, which is mediated by the enzyme cyp27c1, a member of the cytochrome P450 family (Enright et al. 2015). In Nicaraguan Midas cichlids, expression of *cyp27c1* is correlated with A1 and A2 based chromophore usage (Torres‐Dowdall et al. 2017). This high variability enabled cichlids to tune their visual system to a wide range of light conditions (reviewed in Carleton et al. 2016).

Several Neotropical cichlid species from Nicaragua have concurrently colonized novel photic environments (Fig. 1) and, hence, prove ideal for studying convergent evolution of the visual system. These fishes inhabit the same environments; many rivers, two old great lakes (Lakes Nicaragua and Managua) and a number of crater lakes (Villa 1976), but vary substantially in size, habitat preference, trophic level, and coloration (Table S1). The two great lakes are located in the Nicaragua Depression and geological data suggests that the lake basin formed less than 1 Mya (Bussing 1976). Approximately 500,000 years ago (Bussing 1976; Elmer et al. 2010a), cichlids colonized these lakes from adjacent rivers (most likely including Río San Juan and Río Punta Gorda; Fig. 1). More recently and in some cases less than 2000 generations ago, cichlids further colonized numerous, very young (1200–22,000 years; Kutterolf et al. 2007; Pardo et al. 2008) crater lakes from the two great lakes (Elmer et al. 2010a; Elmer et al. 2010b; Kautt et al. 2016; Franchini et al. 2017; Kautt et al., 2018. These environments differ considerably in their light conditions (Torres‐Dowdall et al. 2017); the rivers are shallow and turbidity varies strongly during the year associated with seasonality in precipitation, the great lakes are shallow and constantly turbid, whereas the crater lakes are considerably deeper and the water is much clearer (Cole 1976; Elmer et al. 2010a). As a result, the crater lake photic environment is shifted toward shorter wavelengths compared to the turbid great lakes and rivers (Fig. 1). Accordingly, Midas cichlids (*Amphilophus* cf. *citrinellus*) adaptively shifted visual sensitivity toward absorbing light at shorter wavelengths in crater lakes (Torres‐Dowdall et al. 2017). These changes in cone opsin expression are largely genetically determined (Torres‐Dowdall et al. 2017), but phenotypic plasticity could also contribute to the observed differences among photic environments (Härer et al. 2017).

**Figure 1:**
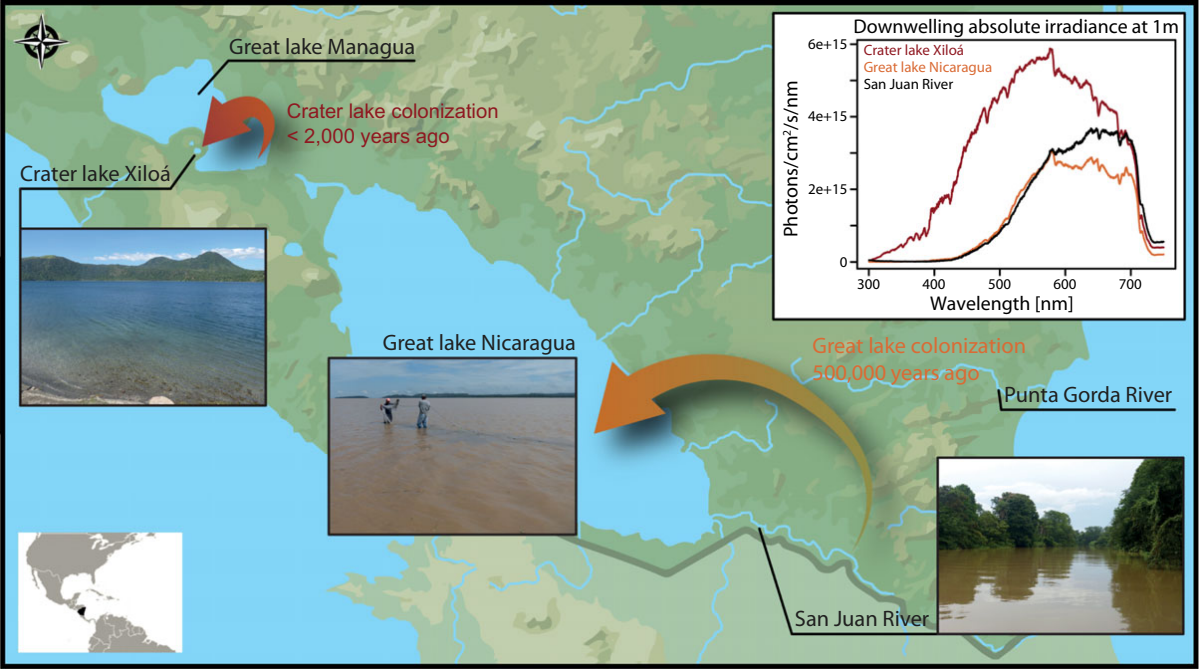
Map of Nicaragua showing all sampling locations including two rivers (Punta Gorda and San Juan), two great lakes (Nicaragua and Managua) and one crater lake (Xiloá). The great lakes were colonized from riverine environments around 500,000 years ago and crater lake Xiloá was colonized from great lake Managua, most likely less than 2000 years ago (Kautt et al. 2016; Franchini et al. 2017). Rivers and great lakes are characterized by shallow and turbid water, crater lakes have much clearer, deeper water, which causes shifts in the ambient photic environment. Absolute irradiance measurements of downwelling light are shown for a depth of one meter (white inset). In the crater lake, overall levels of light are higher but are also more shifted toward shorter wavelengths. Light spectra of the great lake and river are very similar from 300 to 580 nm. At longer wavelengths, more light is present in the river compared to the great lake leading to a longer wavelength shifted light environment in the river.

Since several ecologically distinct cichlid species followed the same colonization route, this natural experiment could help to understand how predictable evolution of the visual system is in response to shared environmental conditions and to what extent it is affected by ecological differences among species. Based on our knowledge on photic environments and on Midas cichlids’ visual system (Torres‐Dowdall et al. 2017), we predicted that cichlids adaptively changed their visual sensitivities after colonizing novel photic environments. However, unique demographic histories and differences in standing genetic variation of the source populations impede making reliable predictions concerning convergence of the underlying molecular mechanisms. Still, we further predicted that rapid adaptation occurred predominantly via regulatory changes. To this end, we analyzed retinal transcriptomes from three populations of seven cichlid species using high‐throughput RNA sequencing (RNA‐Seq) and further analyzed coding sequences of genes involved in color vision to explore structural and regulatory variation.

## Methods

### SAMPLE COLLECTION

We investigated the visual system of the following Neotropical cichlids from Nicaragua: *Amatitlania siquia*, *Archocentrus centrarchus*, *Astatheros rostratus*, *Hypsophrys nematopus*, *Hypsophrys nicaraguensis*, and *Parachromis managuensis*. Recently, quantitative Real‐Time PCR (qPCR) and *in situ* hybridization analyses showed that crater lake Midas cichlids (*Amphilophus* cf. *citrinellus*) have a short wavelength shifted visual system compared to one source population from the great lakes (Torres‐Dowdall et al. 2017). Thus, we included *A. sagittae* from Lake Xiloá and *A. citrinellus* from Río San Juan and Lake Managua to validate that different molecular techniques yield similar results (Fig. S1). Another species of Midas cichlids, *A. astorquii* from Lake Apoyo, was not part of the focal analysis but was included since it shows similar cone opsin expression patterns as *A. siquia* from Lake Xiloá (Fig. S3). Generally, 5–6 specimens per species and sampling location (except for *A. centrarchus* from the Great lake with n = 3) were obtained from turbid rivers (Río San Juan and Río Punta Gorda), turbid great lakes (Lakes Managua and Nicaragua) and a clear crater lake (Lake Xiloá; Fig. 1 and Table S2). Many cichlids undergo ontogenetic changes in cone opsin expression (Carleton et al. 2008; Härer et al. 2017), hence, only adult fish were collected. Fish were caught using gill nets at depths between 0 and 5 meters. Nets were regularly checked and alive specimens were removed from the nets and immediately sacrificed by applying an overdose of MS‐222 and subsequent cutting of the vertebral column. Retinas were dissected and stored in RNAlater (Sigma‐Aldrich, St. Louis, Missouri) until RNA extraction. To reduce diurnal variation in opsin expression, retinas were dissected only in bright daylight conditions (between 10 am and 2 pm). All samples were collected during field expeditions in 2013 and 2015 (under MARENA permits DGPN/DB‐IC‐004‐2013 & DGPN/DB‐IC‐015‐2015). Further, absolute irradiance was measured for each environment at a depth of 1 meter, using an Ocean Optics FLAME‐S‐XR1‐ES spectrometer and a cosine corrector. Conversion from watts to photons is based on calculations from Johnsen (2011).

### LIBRARY PREPARATION AND ILLUMINA SEQUENCING

RNA was extracted using the commercial RNeasy Mini Kit (Qiagen, Hilden, Germany) and concentrations were measured on a Qubit Fluorometer (Thermo Fisher Scientific, Waltham, Massachusetts). RNA integrity was determined (Agilent 2100 Bioanalyzer; Agilent Technologies, Santa Clara, California), all samples had RIN values above 6. Libraries were generated using the TruSeq Stranded mRNA HT sample preparation kit (Illumina, San Diego, California) according to the manufacturer's protocol. The final libraries were amplified using 15 PCR cycles, quantified on a Qubit Fluorometer and quality was assessed (Agilent 2100 Bioanalyzer). All samples were individually labeled with unique barcodes and 30 ng of each sample were pooled and paired‐end sequenced in two lanes of the Illumina flow cell (2 × 150 bp) on an Illumina HiSeq2500 platform at TUCF Genomics (Tufts University, Medford, Massachusetts).

### QUALITY CONTROL, TRANSCRIPT ASSEMBLY, AND MAPPING

A total of 246,322,577 reads were collected (median: 1,410,687 reads per individual; Table S3). Illumina adapters were removed and reads were trimmed with Trimmomatic v0.36 (Bolger et al. 2014). For opsin expression analyses, trimmed reads were mapped against *A. citrinellus* reference sequences of 50 bp of the 5’ UTR and the first 150 bp of the coding sequence (CDS) for all cone opsins with bowtie 2.3.0 (Langmead and Salzberg 2012). We chose this approach instead of mapping against the full CDS because *rh2aα* and *rh2aβ* underwent gene conversion (Torres‐Dowdall et al. 2017). Only the 5’ UTR and the first exon show sequence variation whereas the rest of the CDS is identical, hence, reads could not be unambiguously assigned to either of the two paralogs for exons 2–5. Overall, mapping against the UTR and the first exon revealed similar results as when mapping against the full CDS. To analyze *cyp27c1* gene expression, reads were mapped against the full CDS from *A. citrinellus* (Torres‐Dowdall et al. 2017) using TopHat2 v2.1.1 (Kim et al. 2013). Bam files were converted into sorted sam files with samtools v1.43 (Li et al. 2009) and count tables were created with HTSeq v0.6.1 (Anders et al. 2015).

### SEQUENCE ANALYSES

Complete CDS of opsin genes consistently expressed at high levels (*sws2a*, *rh2a*β, and *lws*) were obtained by mapping reads against *A. citrinellus* reference sequences (Torres‐Dowdall et al. 2017) with CLC Genomics Workbench 8 (Qiagen, Hilden, Germany). Consensus sequences were generated with a minimum coverage of 10x per locus and the noise threshold was set to 0.35 (i.e., heterozygotes were only scored if the minor allele frequency was > 35%). Nucleotide sequences were aligned using SeaView v4 (Gouy et al. 2010) and translated into amino acid sequences to score nonsynonymous substitutions. Only nonsynonymous substitutions that were variable within species are shown in Table S4, all nonsynonymous substitutions among species are shown in Tables S5–S7. To investigate sites under positive selection, random site models in PAML were used (Yang 2007). We tested for variation in ω (i.e., dN/dS) across sites (M3/M0) and for the presence of positively selected sites (M2/M1) that were identified with Bayes’ Empirical Bayes in PAML (Table S8; Yang 2007).

### GENE EXPRESSION ANALYSES

Gene expression analyses were performed in R v3.2.3 (R Core Team 2015). Cone opsin expression was calculated as the proportion of each cone opsin relative to the overall cone opsin expression using the following equation:

$$\mathrm{Proportional}\;\mathrm{expression}\;{\left(\mathrm{PE}\right)}_{i}=\frac{\mathrm{Read}\;\mathrm{coun}t_{i}}{\mathrm{Read}\;\mathrm{count}{\;}_{\sum \mathrm{all}\;\mathrm{cone}\;\mathrm{osins}}}\;$$

*Read count_i_* represents number of reads for a particular cone opsin. We further calculated predicted sensitivity indices, which provide information on visual sensitivity by integrating the peak of maximum absorption (λ_max_) and proportional expression for each opsin. λ_max_ values are based on Midas cichlids (*sws2b*, *sws2a*, *rh2b*, *rh2a*β, *rh2a*α, & *lws*; Torres‐Dowdall et al. 2017) and Nile Tilapia (*sws1*; Spady et al. 2006):

$$\begin{array}{ccc} \;X_{j} & & =\;\mathrm{PE}{}_{\mathrm{sws}1}\times 360\mathrm{nm}+\mathrm{PE}{}_{\mathrm{sws}2b}\times 425\mathrm{nm}+\mathrm{PE}{}_{\mathrm{sws}2a}\\ & & \;\times \;456\mathrm{nm}+\mathrm{PE}{}_{\mathrm{rh}2b}\times 472+\mathrm{PE}{}_{\mathrm{rh}2a\beta }\times 517\mathrm{nm}\\ & & \;+\;\mathrm{PE}{}_{\mathrm{rh}2a\alpha }\times 527\mathrm{nm}+\mathrm{PE}{}_{\mathrm{lws}}\times 560\mathrm{nm} \end{array}$$

**Figure 2:**
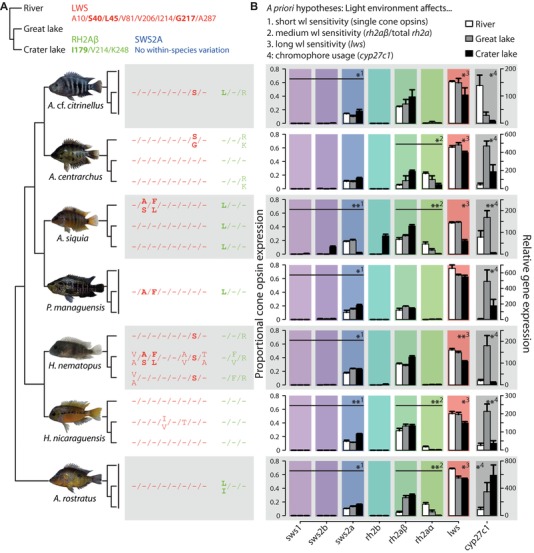
Structural changes of cone opsin genes and regulatory changes of cone opsins and *cyp27c1* associated with colonization of novel photic environments. (A) Amino acid sites variable within species are shown (numbers according to bovine RH1) for LWS (red) and RH2Aβ (green), but none were found for SWS2A (blue). Three of the variable LWS sites and one RH2Aβ site are under positive selection (bold). Phylogenetic relationships among species are based on a phylogeny from Lopez‐Fernandez et al. (2010). Note that the relationships among populations within each species are solely inferred based on our knowledge about the colonization history of Nicaraguan lakes. (B) Species shift visual sensitivity among environments by differential expression of varying genes, including changes in proportional expression of single cone opsins (1), ratio between shorter and longer green‐sensitive opsins (*rh2a*β and *rh2a*α; 2), proportional expression of the red‐sensitive *lws* (3), and *cyp27c1* expression (4). These four measures were tested separately by comparing gene expression across all three populations of each species using Kruskal–Wallis tests. Significant differences in gene expression indicate that, overall, the visual system is affected by environment (^*^ *P* < 0.05, ^**^ *P* < 0.01, FDR corrected). wl = wavelength.

**Figure 3:**
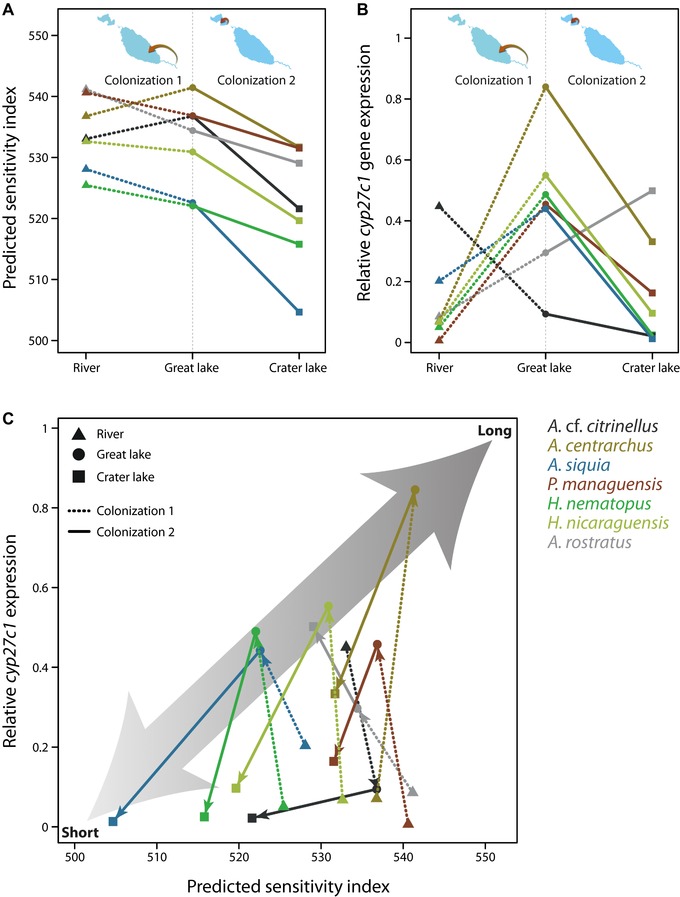
Changes in predicted sensitivity index (A) and relative *cyp27c1* expression (B; note that this measure differs from Fig. 2, see Methods for more details) for both colonization events. Briefly, the predicted sensitivity index takes into account proportional expression and peaks of maximum absorption (see Methods for more details on calculation) and provides information on predicted visual sensitivity. *cyp27c1* expression is indicated relative to the highest value for each species. Predicted sensitivity index is affected by environment (*P* < 0.001) and by species identity (*P* = 0.026) showing convergent changes among species. Differences among species are maintained (based on Spearman's rank correlation coefficient) after colonizing the crater lake (ρ = 0.786, *P* = 0.036) but only suggested after colonizing the great lake (ρ = 0.679, *P* = 0.094). For expression of *cyp27c1*, only the species by environment interaction term was significant (*P* = 0.03), suggesting that there is no convergence among species. Along the same line, we could not detect evidence for maintenance of ranks after colonizing novel environments. When integrating predicted sensitivity index and *cyp27c1* expression (C), parallel changes across environments can be observed for most species. Lower values on both axes represent visual sensitivities at shorter wavelengths. For each species, vectors connect mean values for populations from three environments (sample sizes are indicated in Table S2). Distances between direction and length of vectors were measured for both colonization events separately and combined. Actual data was compared to a random set of 999 permutations to obtain significance values. Significant parallelism in the direction of change was detected for colonization 2 (*P* = 0.041) but also when both colonization events were combined (*P* = 0.037).

*PE* is the proportional expression of each cone opsin in specimen *j*. This equation is adapted from Carleton et al. (2016), but was modified to incorporate single and double cone opsin expression in one index. Relative expression of *cyp27c1* was measured either as number of reads mapped to the *cyp27c1* coding sequence per one million reads (Fig. 2B) or relative to the highest expression value of *cyp27c1* within each species to reduce among‐species variation in overall expression levels to better illustrate convergence (Fig. 3).

Regarding cone opsin expression, the photic environment could affect visual sensitivity in the short, medium, and long parts of the light spectrum, hence, we hypothesized that (i) total opsin expression comprised by single cone opsins, (ii) proportions of the green‐sensitive *rh2aβ* and *rh2aα*, in case that both paralogs of *rh2a* were expressed (as shown in *A. ocellatus*; Escobar‐Camacho et al. 2017), and (iii) proportional expression of the red‐sensitive *lws* (as seen in Torres‐Dowdall et al. 2017) would be affected. Accordingly, we tested for effects of species, photic environment, and their interaction for these different aspects of cone opsin expression (single cone opsin expression, *rh2aβ/rh2aα* ratio, *lws* expression), as well as for predicted sensitivity indices and *cyp27c1* expression. For this, we used Scheirer‐Ray‐Hare tests, a nonparametric equivalent to two‐way ANOVA (Scheirer et al. 1976). Further, we tested the above‐mentioned three a priori hypotheses separately for each species using non‐parametric Kruskal–Wallis tests.

Following Stuart et al. (2017), we tested for parallelism of color vision across species associated with colonization of novel photic environments. First, we produced two dimensional vectors for each species, connecting mean values of populations (sample sizes are shown in Table S2) from the three environments by taking into account cone opsin expression (predicted sensitivity index) and *cyp27c1* gene expression (Fig. S2). We calculated pairwise differences between mean angles and lengths of vectors among all species for both colonization events (colonization 1: river‐great lake, colonization 2: great lake‐crater lake) separately. In total, this added up to 21 pairwise tests and next, we calculated the sums of all pairwise differences. Note that we tested for parallelism for each colonization event separately but also for both colonization events combined. For the latter, we added the sums of all pairwise differences from both colonization events. We then randomized species identity within each environment and created 999 datasets with vectors connecting one individual from the ancestral environment (river or great lake in colonization 1 or 2, respectively) and the derived environment (great lake or crater lake in colonization 1 or 2, respectively). Within each randomized dataset, we calculated differences between angles and lengths as described above. In total, we performed 21 pairwise comparisons to obtain the same number of comparisons as in our original dataset. We added all sums of pairwise differences within each dataset and determined in how many cases the sums in the permuted datasets were smaller compared to our actual data. Statistical significance was determined at the .05 level.

## Results

The aquatic landscape of Nicaragua represents an intriguing setting, where seven ecologically differentiated species of cichlid fishes have concurrently colonized two novel photic environments (Fig. 1 and Table S1). Color vision is particularly variable in cichlids and is mediated by visual pigments located in photoreceptor cells of the retina, which are composed of a light‐absorbing chromophore that is covalently bound to a transmembrane opsin protein (Wald 1968; Yokoyama 2000; Ebrey and Koutalos 2001). In aquatic vertebrates, visual sensitivity is mainly determined by three molecular mechanisms: chromophore usage (indirectly measured as *cyp27c1* gene expression, see Methods for more details), as well as structural changes or differential expression of opsin genes (Terai et al. 2006; Hofmann et al. 2009; O'Quin et al. 2010; Torres‐Dowdall et al. 2017). Our analyses identified divergence in these three mechanisms among populations of the same species (sample sizes are shown in Table S2) for all seven species (Fig. 2).

We obtained complete CDS of *sws2a*, *rh2aβ*, and *lws* from all species since these genes were consistently expressed at high levels (Fig. 2B). Overall, we found ten (*sws2a*), seven (*rh2aβ*), and 18 (*lws*) nonsynonymous substitutions among species (Tables S5–S7). Out of those, only two (*rh2aβ*) and six (*lws*) varied among populations within species, but none in *sws2a* (Fig. 2A and Table S4). As we were interested in changes associated with the colonization of novel photic environments, we focused on amino acid substitutions that varied among populations of the same species. All, but one, of these nonsynonymous substitutions were located in transmembrane regions that are important for functional dynamics and spectral tuning of photopigments (Asenjo et al. 1994; Hunt et al. 2001; Carleton et al. 2005; Seehausen et al. 2008; Hofmann et al. 2009). The number of variable amino acid residues differed strongly among species (Fig. 2A and Table S4). Analyses of molecular evolution suggest that of those residues varying among populations, one (RH2Aβ) and three (LWS) were under positive selection (Table S4, gray boxes); but in total, nine residues were found to be under selection in LWS (Table S8). Two of these LWS residues were shared among *A. siquia* and *H. nematopus* (S40A and L45F; Table S4). However, these showed fixed differences between river and both crater and great lakes in *A. siquia* (Fig. 2A), but in *H. nematopus* these were polymorphic in the great lake and fixed for the same allele in river and crater lake. Hence, there was no convergence in any of the species after colonizing novel photic environments (Fig. 2A).

Since there were no convergent changes in structural variation associated with photic environments, we focused on the other main axes by which visual sensitivity can be altered (opsin expression and chromophore usage). Most specimens, independent of habitat of origin, expressed a long wavelength sensitive cone opsin subset (*sws2a*, *rh2a*β/α, and *lws*; sensu Carleton et al. 2016; Fig. 2B). This expression pattern is likely well‐suited to the predominantly long wavelength shifted light conditions of turbid large rivers, like the San Juan river, of the Neotropics (Escobar‐Camacho et al. 2017). The only exception to this pattern was the population of *A. siquia* from the crater lake, that expressed the violet‐sensitive *sws2b* instead of the blue‐sensitive *sws2a* in single cones and the blue‐green sensitive *rh2b* in double cones (Fig. 2B). By using quantitative real‐time PCR, we recently found that similar changes occurred in one Midas cichlid species, *A. astorquii*, from another crater lake (Torres‐Dowdall et al. 2017), and here we confirmed these results using RNA‐Seq (Fig. S3). These two species were the only ones that expressed a different subset of opsin genes as adults, which changed predicted visual sensitivity toward the short wavelength shifted photic environment of the crater lake (Fig. 3).

Most species shifted gene expression of cone opsins toward absorbing light at shorter wavelengths following the two colonization events, particularly in the clear water crater lake, but the exact differentially expressed genes were not necessarily shared among all species. We specifically tested whether species changed expression of short (total expression of single cone opsins), medium (*rh2aβ*/*rh2aα* ratio), or long (*lws* expression) wavelength sensitive opsins. Across all species, proportional expression of short wavelength sensitive opsins commonly increased after colonization of the crater lake and varied among environments in a way that depended on species identity (Scheirer‐Ray‐Hare test, species x environment interaction term *P* < 0.001). This significant interaction can most likely be explained by deviating expression patterns in *A. centrarchus*, which did not change expression, and in *A. siquia*, which decreased the overall proportional single cone opsin expression in the crater lake, but switched from expressing *sws2a* to *sws2b* (Fig. 2B). Expression ratio of the green‐sensitive *rh2a* paralogs differed significantly among environments (*P* < 0.001) and species (*P* = 0.014). However, only *A. centrarchus*, *A. siquia*, *H. nicaraguensis*, and *A. rostratus* showed substantial expression of both *rh2a* paralogs and significant changes in *rh2aβ*/*rh2aα* ratio. Whereas in *H. nicaraguensis*, *rh2aα* was only expressed in the riverine population, the other three species gradually decreased expression of the longer wavelength sensitive *rh2aα* from river to great lake to crater lake (Fig. 2B and Fig. S4). Expression of the red‐sensitive *lws* differed among environments (*P* < 0.001) and species (*P* = 0.011) and all species, except for *P. managuensis*, changed *lws* expression (Fig. 2B). In general, *lws* expression was consistently lowest in the crater lake and the most pronounced changes occurred after colonization of the crater lake (except for *A. rostratus* where *lws* expression was strongly altered only after colonizing the great lakes). While most species showed similarities in the direction of gene expression changes, it should be noted that the magnitude of change varied in some cases (e.g., compare *lws* expression of *A. centrarchus* and *A. siquia*).

Rearing fish of species that naturally occur in the great lake and the crater lake under common laboratory conditions revealed that differences in cone opsin expression patterns were largely genetically determined, whereas rearing environment only had minor effects on expression patterns, but was variable among species (Fig. S5 and Fig. S6). The variation in *cyp27c1* gene expression (i.e., chromophore usage) was affected by the environment but depended upon species identity (interaction term *P* = 0.03; Fig. 3B). In general, expression of *cyp27c1* was lower in rivers compared to the turbid great lake (except for *A*. cf. *citrinellus*) and higher in the turbid great lake compared to the clear crater lake (except for *A. rostratus*; Fig. 2B). This suggests changes in A1 to A2 ratios, with higher A2 usage in the great lake compared to both the crater lake and riverine populations, resulting in sensitivities of the individual visual pigments shifted toward longer wavelength in the former compared to the latter environments.

To integrate overall opsin gene expression and to predict shifts in visual sensitivity, we calculated predicted sensitivity indices (see Methods), which were significantly affected by photic environment (*P* < 0.001) and by species identity (*P* = 0.026), but not by their interaction (Fig. 3A). As stated above, for expression of *cyp27c1*, only the species x environment interaction was significant (Fig. 3B). To determine levels of convergent change in color vision, we integrated cone opsin expression (predicted sensitivity index) and *cyp27c1* expression. We performed vector analyses and specifically tested for parallelism across species in direction (vector angles, γ) and magnitude (vector lengths, ΔL) of phenotypic change (Fig. 3C). When considering both colonization events combined, we detected parallelism in direction (γ, *P* = 0.037) but not in magnitude (ΔL) of phenotypic change among all species (Fig. 3C). When investigating each colonization event separately, parallel changes were only found for the direction of change during colonization of the crater lake (*P* = 0.041). Notably, for each colonization event, only six species appeared to show parallelism and one species (*A*. cf. *citrinellus* for colonization 1 and *A. rostratus* for colonization 2) deviated from this general pattern (Fig. 3). Within the same environment, species varied substantially in predicted sensitivity and these differences and the rank order of the species were maintained after colonizing the crater lake (Spearman's rank correlation coefficient; ρ = 0.786, *P* = 0.036) but only suggested after colonizing the great lake (ρ = 0.679, *P* = 0.094; Fig. 3A).

## Discussion

Convergent evolution is recognized as compelling evidence for natural selection and has been observed in many traits from a diverse set of organisms (Reznick et al. 1996; Rundle et al. 2000; Mahler et al. 2013; Rosenblum et al. 2017), including cichlids (Barluenga and Meyer 2004; Elmer et al. 2010a). However, the exact environmental parameters promoting evolutionary change and the adaptive value of phenotypes, as well as the underlying molecular mechanisms, often remain elusive (Barrett and Hoekstra 2011; Orgogozo 2015). The vertebrate visual system overcomes some of these uncertainties since there is a good understanding of how molecular changes in coding regions as well as modified expression patterns of opsin genes affect visual phenotypes and how phenotypic differences are shaped by the photic environment (Yokoyama 2000; Bowmaker 2008; Hofmann et al. 2009; Ryan and Cummings 2013; Cronin et al. 2014; Enright et al. 2015; Marshall et al. 2015; Carleton et al. 2016). The natural experiment in Nicaraguan lakes where several cichlid species concurrently colonized the same two novel photic environments allows us to test whether these species show a similar evolutionary response due to shared selection pressures in novel environments.

Over short‐time scales, for example after the recent colonization of the crater lake, only 1300–1700 generations ago for *A*. cf. *citrinellus* and *A. centrarchus* (Kautt et al. 2016; Franchini et al. 2017), selection is assumed to have acted predominantly on standing genetic variation rather than on *de novo* mutations (Innan and Kim 2004; Barrett and Schluter 2008). Assuming that regulatory regions are under fewer constraints than coding regions (Stern and Orgogozo 2008; Ghalambor et al. 2015), we would expect more variation in gene expression. Hence, especially after crater lake Xiloá was colonized, most adaptive changes were expected to be regulatory. Indeed, we found only few structural changes across localities and none that were convergent among species (Fig. 2A). Note that the potential phenotypic effects of these substitutions (i.e., changes in peaks of maximum absorption) were not evaluated in this study but are a matter of future research. Expression of cone opsins (functional under bright light and responsible for color discrimination) and *cyp27c1* (conversion of vitamin A1 to A2 derived chromophore; Enright et al. 2015) substantially varied among populations in all species and often changed in the same direction (Fig. 3). After colonizing the crater lake from the great lake, changes occurred in the direction predicted based on environmental differences and knowledge on the visual system of Midas cichlids (Fig. 1; Torres‐Dowdall et al. 2017). For the colonization of great lakes from rivers, cone opsin expression differed in a way that resulted in predicted visual sensitivities slightly shifted toward shorter wavelengths that fits the differences in light conditions (compare Fig. 1 to Fig. 3A). However, changes in *cyp27c1* expression suggest that sensitivity of the resultant visual pigments would be shifted toward longer wavelengths in the great lake compared to the riverine populations (Fig. 3B). Nonetheless, the changes in cone opsin and *cyp27c1* expression occurred in the same direction in all species (but *A*. cf. *citrinellus*), hence, we interpret these to be adaptations to the light conditions of the great lakes (Fig. 3). Microspectrophotometry will be required to determine the overall effects of changes in *cyp27c1* expression on visual sensitivity.

Recent studies have shown that phenotypic convergence can be mediated by the same (Zhen et al. 2012; Projecto‐Garcia et al. 2013) or different (Natarajan et al. 2016; Castiglione et al. 2018) molecular mechanisms. We found that across our study species, varying subsets of cone opsin genes (as well as *cyp27c1*) were differentially expressed among the three environments, that is adjustment of the visual system to new environments occurred by changing expression of single cone opsins, the two green‐sensitive *rh2a* genes and/or the red‐sensitive *lws*, as well as *cyp27c1* (Fig. 2B). Despite these differences among species, changes in predicted visual sensitivity occurred in a convergent manner, suggesting that phenotypic change can be produced by different molecular routes in our system. However, these different routes might result in phenotypic differences that are not reflected in the model used to predict sensitivity. The observed differences among species could be explained by the independent demographic histories and, hence, differences in standing genetic variation, as seen in some of our study species (Elmer et al. 2013; Franchini et al. 2017). On the contrary, shared genetic variation could also promote convergence among closely related species by constraining phenotypic evolution (Haldane 1932; Futuyma et al. 1995; Schluter 1996). However, we argue that in our system, convergent evolution is mainly driven by shared selection pressures rather than genetic constraints since species have long divergence times (5–15 million years (my) based on Hulsey et al. 2010; 13–55 my based on Lopez‐Fernandez et al. 2013) and expression patterns varied within monophyletic groups but were shared among more distantly related species (e.g., *rh2aβ* to *rh2aα* ratio; Fig. 2B and Fig. S4).

The largest shift in cone opsin expression, resulting in the shortest wavelength sensitive visual phenotype, was observed in *A. siquia* from crater lake Xiloá (Fig. 3). This population deviated from the common subset of expressed cone opsins (*sws2a*, *rh2aβ*/*α*, *lws*) and additionally expressed opsins sensitive to shorter wavelengths, *sws2b* and *rh2b*, in single and double cones, respectively (Fig. 2B). Similar results were found in Midas cichlids from another crater lake (Torres‐Dowdall et al. 2017). This pattern of expressing more than three cone opsins at the same time differs from what is commonly observed in African cichlids (reviewed in Carleton et al. 2016), but see (Sabbah et al. 2010), suggesting that adaptive evolution of the visual system might be produced by different mechanisms in these cichlid lineages. Crater lake populations of *A. siquia* and *A*. cf. *citrinellus* not only changed the proportions of cone opsins, but also expressed a different subset of cone opsins as adults compared to the other cichlid species investigated in this study. This nicely illustrates that convergent phenotypic evolution occurred via different routes, as also suggested for adaptive evolution of the dim‐light sensitive rhodopsin (Castiglione et al. 2018). However, at this point, we are still lacking knowledge about the underlying molecular bases for the observed gene expression differences that produced the convergent changes in visual sensitivity. However, QTL analyses in African cichlids have shown that opsin expression is regulated largely independently (Carleton et al. 2010; O'Quin et al. 2011; Nandamuri et al. 2017a). Based on these results, we conclude that convergent evolution of visual sensitivity in our system is brought about by different molecular mechanisms (i.e., differential expression of varying sets of genes), which might further have distinct genetic bases, a hypothesis that is currently under investigation.

The expression changes seen in all species across localities most likely represent adaptations to the respective photic environments but the question remains why differences among species are maintained within each environment. Many factors are associated with the great diversity of structural and regulatory changes of cone opsins seen in African cichlids, among which species‐specific ecology appears to play a very important role (Hofmann and Carleton 2009; O'Quin et al. 2010; Irisarri et al. in press). The Nicaraguan cichlids investigated in our study differ considerably in body size, trophic level, habitat use, coloration (Table S1), and in population size and genetic diversity (Elmer et al. 2013; Franchini et al. 2017). Still, almost all species evolved patterns of gene expression resulting in similar changes of predicted visual sensitivity, providing evidence for common selection pressures acting on the visual system. This convergence suggests that the overall photic environment shapes visual sensitivity, which is in line with results from other organisms (Cronin et al. 2014; Marshall et al. 2015). Yet, within each environment we found that cone opsin expression differed remarkably among species and these differences were maintained across environments (Fig. 3A). For instance, *H. nematopus* and *P. managuensis*, two species with extremely different ecologies (Table S1), changed visual phenotypes in parallel across river, great lake, and crater lake in a way that maintained interspecific differences in each of these environments (Fig. 3C). Notably, even though both species shifted their vision to absorb more short wavelength light in the crater lake compared to the river, the riverine population of *H. nematopus* still has a shorter wavelength shifted sensitivity than *P. managuensis* from the crater lake. Taken together, these results strongly suggest that overall photic environment and ecological characteristics of the species both influence color vision, which is in agreement with existing knowledge on adaptive evolution of the visual system (Cronin et al. 2014).

The observed phenotypic differences within species across environments could potentially be produced by phenotypic plasticity since the visual system is plastic in some species of African and Neotropical cichlids, particularly during early development (Hofmann et al. 2010; Dalton et al. 2015; Härer et al. 2017; Nandamuri et al. 2017). However, opsin gene expression has a strong genetic basis in Midas cichlids as differences in natural populations are maintained under common garden conditions (Torres‐Dowdall et al. 2017). To better understand the role of phenotypic plasticity in producing the patterns of gene expression observed in wild‐caught fish, we raised specimens of a subset of our study species (*A*. cf. *citrinellus* and *A. centrarchus* from the great lake, and *A. siquia* and *H. nematopus* from the crater lake but none from the river) in a common laboratory environment. Although we observed varying, but overall low, levels of phenotypic plasticity, cone opsin expression patterns observed in the wild were largely maintained under common rearing conditions (e.g., the unique expression of *sws2b* and *rh2b* in crater lake *A. siquia* was maintained when specimens where reared in the laboratory; Fig. S5). Moreover, specimens from the same habitat of origin clustered together and variation in cone opsin expression was for the main part explained by species identity (Fig. S6). Taken together, these results suggest that phenotypic plasticity might have contributed to some of the observed variability, but current evidence suggests that visual system divergence has a strong genetic component and evolved after colonizing Nicaraguan lakes.

## Conclusion

Coming back to Gould's thought experiment of “replaying life's tape” (Gould 1989), how predictable is evolution after all? Gould argued that contingency would be dominant, which might hold true across long time scales, but at short‐time scales evidence is accumulating that evolutionary change can indeed be predictable, particularly when natural selection is strong and there is reasonable knowledge on the nature of this selection (Reznick et al. 1996; Rosenblum 2006; Elmer et al. 2014; Hendry 2017; Losos 2017; Nosil et al. 2018). The visual system of Nicaraguan cichlids represents an extraordinary model for studying adaptive evolution as lakes differ markedly in their photic environments and we have a very good understanding of the colonization history of these lakes. Here, we present a natural experiment where after sequentially colonizing two novel photic environments, changes in expression levels of different genes affecting the visual system resulted in rapid and convergent changes of predicted visual sensitivity of seven cichlid species. Further, cone opsin expression differences among species appeared to be maintained after colonizing new environments, emphasizing on the important role of species’ ecology in shaping visual systems. In sum, based on our results and knowledge on the visual system of cichlids, we argue that these changes (i) are, at least partially, genetically determined, (ii) represent an example of convergent phenotypic adaptation to the prevalent photic environments and (iii) are produced by nonconvergent changes at the molecular level (i.e., expression changes of different genes that constitute visual pigments).

## DATA ACCESSIBILITY

Raw transcriptomic read data will be deposited in the European Nucleotide Archive.

Associate Editor: Z. Gompert

## Supporting information


**Figure 1**: Proportional expression of six cone opsins based on quantitative Real‐Time PCR (qPCR; y‐axes) and RNA‐Seq (x‐axes) data.
**Figure 2**: Hypothetical data of two species to illustrate our vector analysis for convergent evolution.
**Figure 3**: As previously shown with quantitative Real‐Time PCR (Härer et al. 2017; Torres‐Dowdall et al. 2017), Midas cichlids (*A. astorquii*) from crater lake Apoyo (Top Row) express *sws2b* and *rh2b*, similar to A.
**Figure 4**: Expression ratio of green‐sensitive paralogs (*rh2a*β/total *rh2a*) differed among habitats in all four species that expressed both paralogs (*A. centrarchus*, *A. siquia*, *H. nicaraguensis* and *A. rostratus*).
**Figure 5**: Proportional expression values for all seven cone opsins of wild‐caught specimens from river, great lake and crater lake (white, grey and black bars) as well as laboratory‐reared specimens (orange bars).
**Figure 6**: Principal component analyses was performed using the *prcomp* function of the stats package in R v3.2.3 (R Core Team 2015).
**Table 1**: Morphological and ecological features of all study species.
**Table 2**: Sampling locations and sample sizes for all species.
**Table 3** (provide in a separate Excel sheet): Total number of raw reads, reads mapped to the Midas cichlid reference genome and reads mapped to each cone opsin and *cyp27c1*.
**Table 4**: Variable sites within species leading to amino acid substitutions in RH2Aβ and LWS opsin proteins.
**Table 5**: Variable SWS2A residues across species.
**Table 6**: Variable RH2Aβ residues across species.
**Table 7**: Variable LWS residues across species.
**Table 8**: LRT of positive selection (random sites model in PAML) for three cone opsin coding sequences.

Supporting information
