# Supplementary material for: Convergent phenotypic evolution of the visual system via different molecular routes: How Neotropical cichlid fishes adapt to novel light environments
Source: Evol Lett. 2018 Jul 17;2(4):341–54. doi: 10.1002/evl3.71 (PMC6121847; doi:10.1002/evl3.71)
Supplement: Supplementary file 1 — Figure 1: Proportional expression of six cone opsins based on quantitative Real‐Time PCR (qPCR; y‐axes) and RNA‐Seq (x‐axes) data. Figure 2: Hypothetical data of two species to illustrate our vector analysis for convergent evolution. Figure 3: As previously shown with quantitative Real‐Time PCR (Härer et al. 2017; Torres‐Dowdall et al. 2017), Midas cichlids (A. astorquii) from crater lake Apoyo (Top Row) express sws2b and rh2b, similar to A. Figure 4: Expression ratio of green‐sensitive paralogs (rh2aβ/total rh2a) differed among habitats in all four species that expressed both paralogs (A. centrarchus, A. siquia, H. nicaraguensis and A. rostratus). Figure 5: Proportional expression values for all seven cone opsins of wild‐caught specimens from river, great lake and crater lake (white, grey and black bars) as well as laboratory‐reared specimens (orange bars). Figure 6: Principal component analyses was performed using the prcomp function of the stats package in R v3.2.3 (R Core Team 2015). Table 1: Morphological and ecological features of all study species. Table 2: Sampling locations and sample sizes for all species. Table 3 (provide in a separate Excel sheet): Total number of raw reads, reads mapped to the Midas cichlid reference genome and reads mapped to each cone opsin and cyp27c1. Table 4: Variable sites within species leading to amino acid substitutions in RH2Aβ and LWS opsin proteins. Table 5: Variable SWS2A residues across species. Table 6: Variable RH2Aβ residues across species. Table 7: Variable LWS residues across species. Table 8: LRT of positive selection (random sites model in PAML) for three cone opsin coding sequences. [file EVL3-2-341-s001.docx]

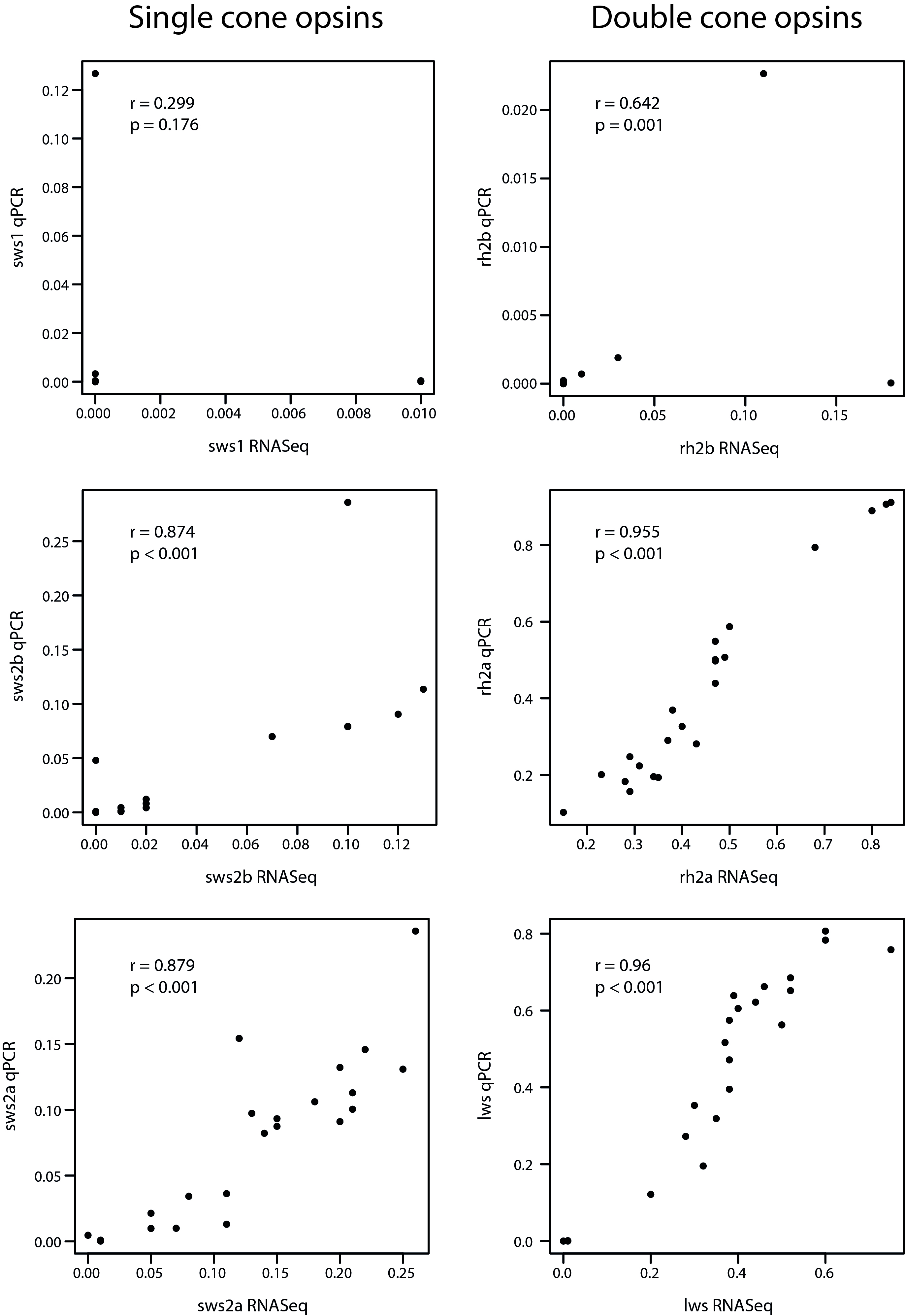


Supplementary Figure 1: Proportional expression of six cone opsins based on quantitative Real-Time PCR (qPCR; y-axes) and RNA-Seq (x-axes) data. All cone opsins with substantial expression levels (particularly the highly expressed sws2a, rh2a and lws) showed strong correlations between the two methods based on Spearman's rank correlation coefficient, thereby validating RNA-Seq as an appropriate method to estimate opsin gene expression levels. Note that expression data of rh2aα and rh2aβ was combined to overall rh2a expression since the two paralogs could not be distinguished by qPCR in previous studies. All qPCR data was obtained from Torres Dowdall et al. (2017).


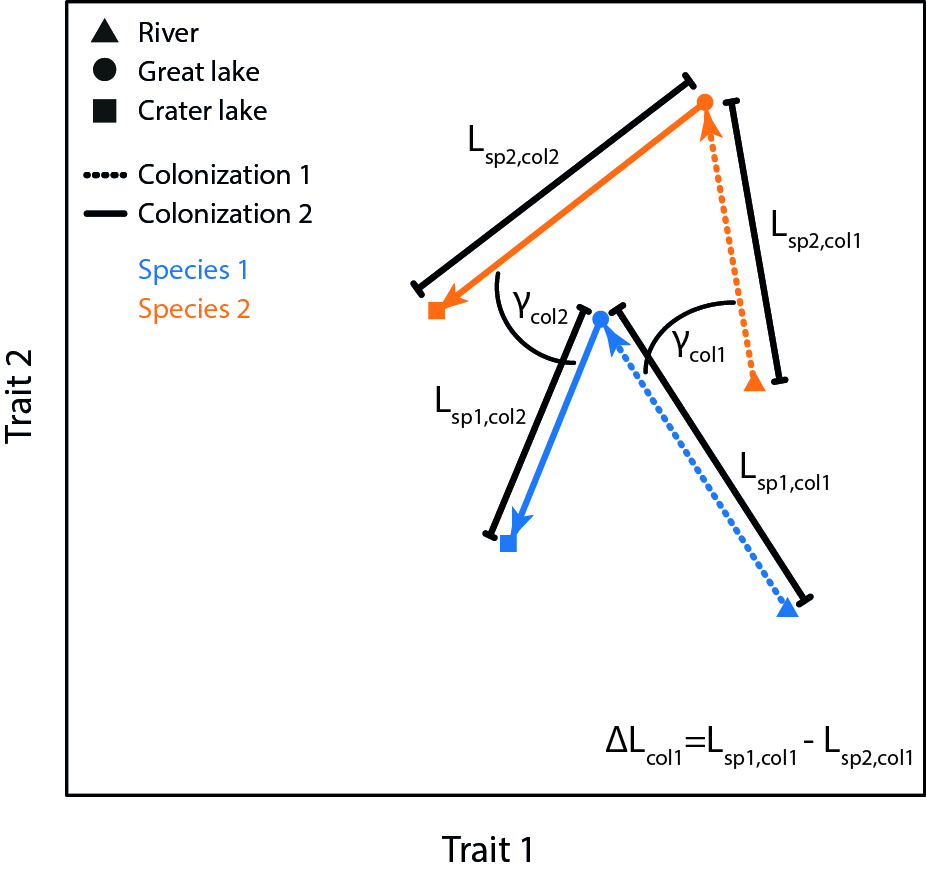


Supplementary Figure 2: Hypothetical data of two species to illustrate our vector analysis for convergent evolution. Two-dimensional vectors connect population means from river, great lake and crater lake for each species. The angle between two vectors is indicated by *γ*. The length of each vector (L) and the pairwise difference in length (*Δ*L) are further shown. *γ* and *Δ*L were calculated for each species pair (total of 21 pairwise comparisons) and each colonization event separately. The sums of these 21 pairwise comparisons were calculated for *γ* and *Δ*L. When both colonization events were analyzed together, the sums of for *γ* and *Δ*L from both colonization events were added. For statistical analyses, species identity was randomized within each habitat and 21 pairwise comparisons of *γ* and *Δ*L were performed. 999 random sets were created and for each, the sums of these pairwise comparisons were added and compared to the original data set. If the sums of the original data set were smaller than 5% of the random data sets, we regarded this as evidence for parallelism.


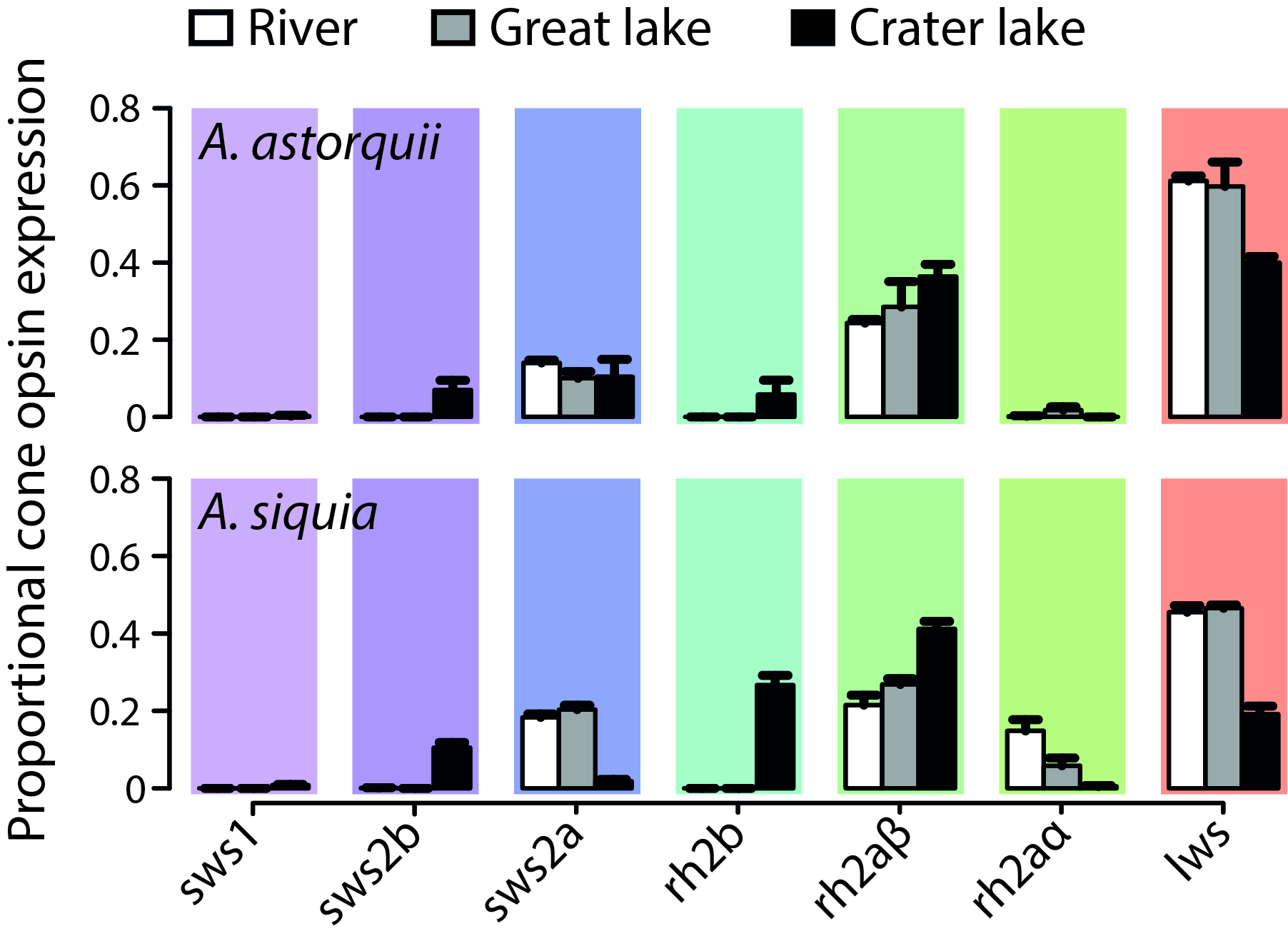


Supplementary Figure 3: As previously shown with quantitative Real-Time PCR (Härer et al. 2017; Torres-Dowdall et al. 2017), Midas cichlids (A. astorquii) from crater lake Apoyo (Top Row) express sws2b and rh2b, similar to A. siquia from crater lake Xiloá (Bottom Row).


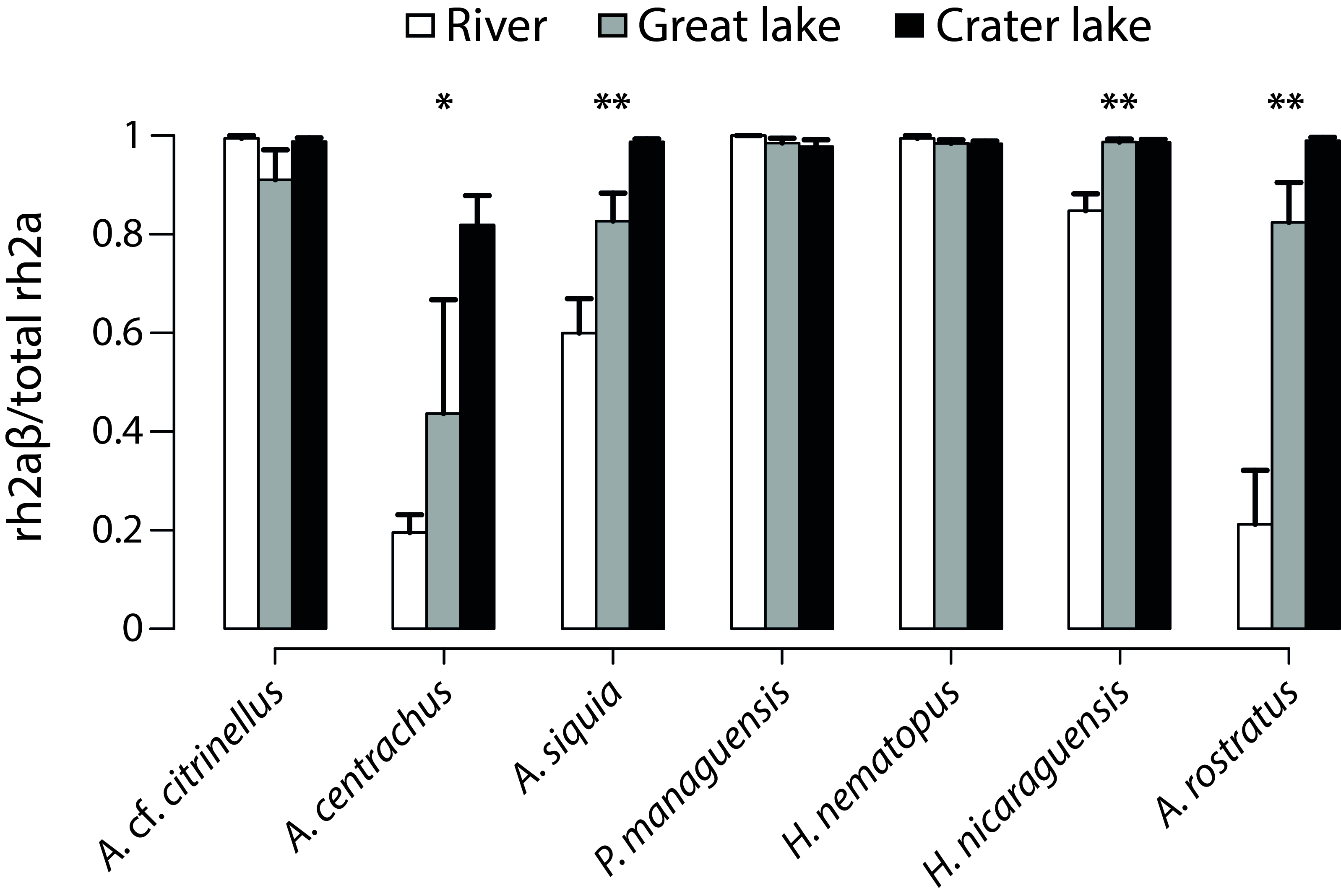


Supplementary Figure 4: Expression ratio of green-sensitive paralogs (rh2aβ/total rh2a) differed among habitats in all four species that expressed both paralogs (A. centrarchus, A. siquia, H. nicaraguensis and A. rostratus). (Kruskal-Wallis test, * p < 0.05, ** p < 0.01, FDR corrected).


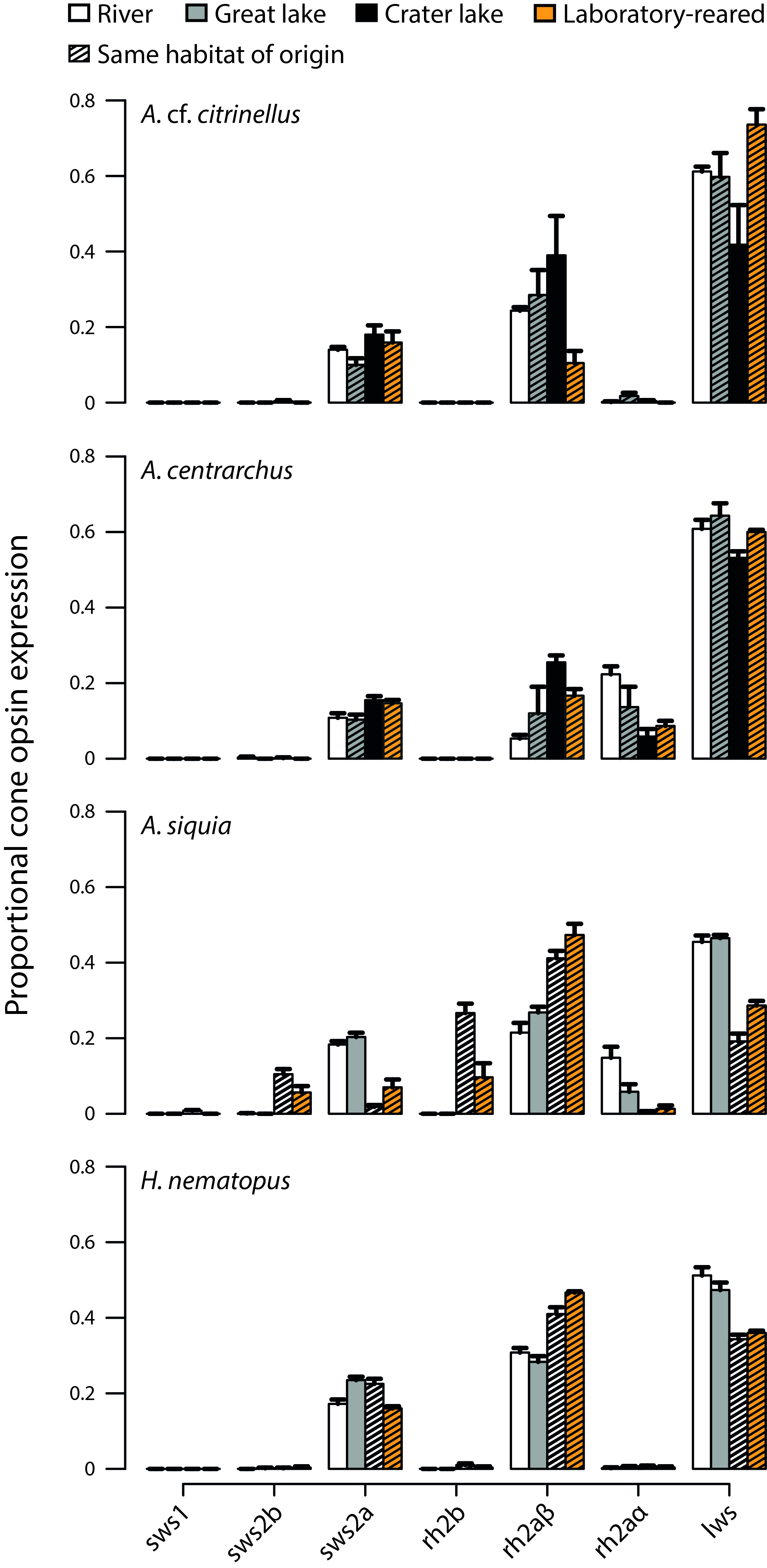


Supplementary Figure 5: Proportional expression values for all seven cone opsins of wild-caught specimens from river, great lake and crater lake (white, grey and black bars) as well as laboratory-reared specimens (orange bars). Hatched bars indicate populations from the same habitat of origin. For A. cf. citrinellus and A. centrarchus, laboratory-reared specimens were originally from the great lake, and from the crater lake for A. siquia and H. nematopus. The extent of phenotypic plasticity apparently varies among species with lower levels in H. nematopus and higher values in A. siquia. This is also reflected by the significant interaction term of species identity and habitat of origin for PC1 scores from the principal component analysis of Fig. S6. Although proportional expression varied in A. siquia, the general set of expressed cone opsins observed in wild-caught specimens (including sws2b and rh2b) was maintained in the laboratory. Note that gene expression data of laboratory-reared specimens of A. cf. citrinellus was generated by a different molecular method (qPCR) and is obtained from Torres Dowdall et al. (2017).


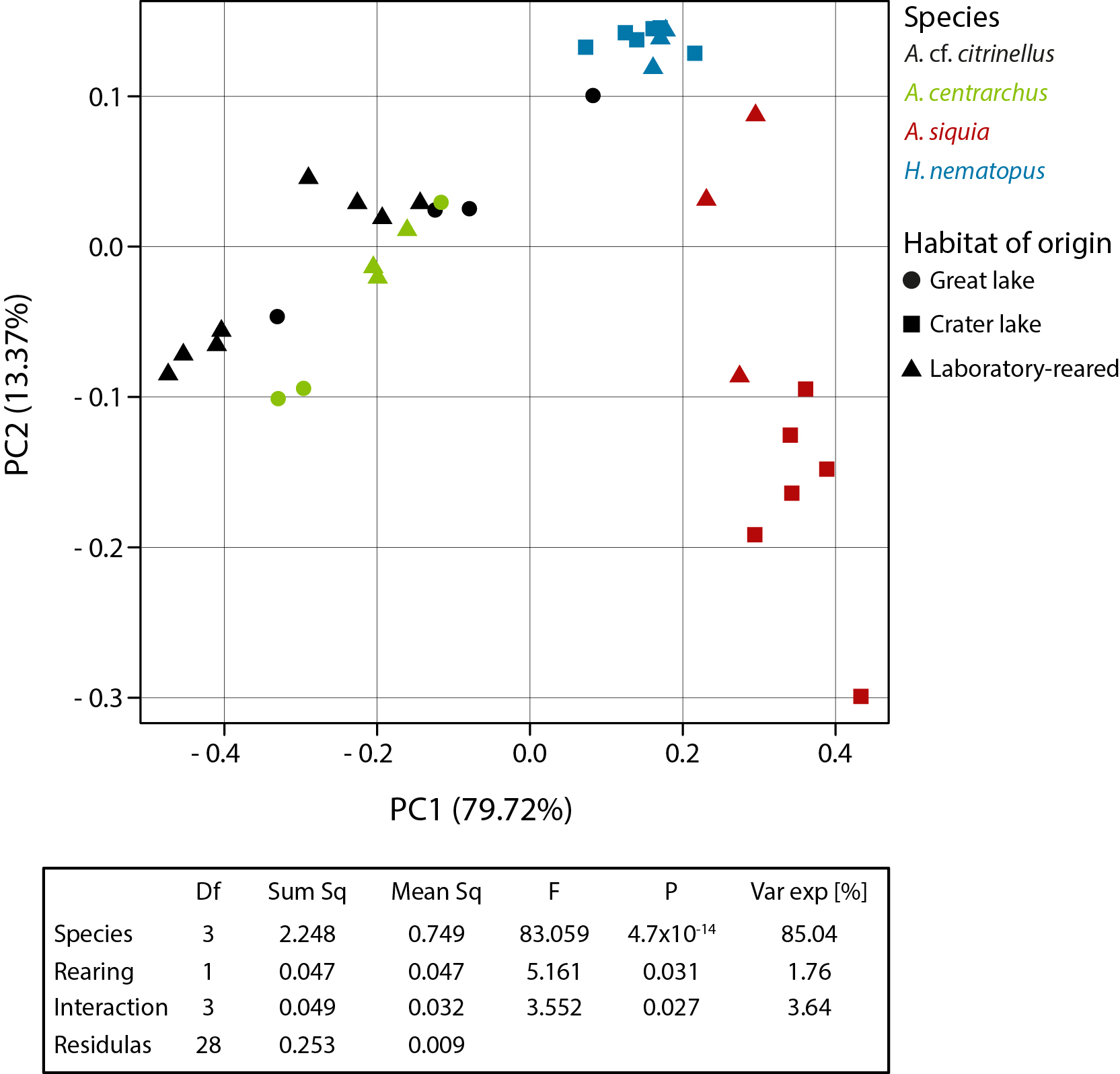


Supplementary Figure 6: Principal component analyses was performed using the *prcomp* function of the stats package in R v3.2.3 (R Core Team 2015). As input, we used proportional cone opsin expression data (see Supplementary Figure 5) of wild-caught specimens (circles and squares) and laboratory-reared specimens (triangles) from the same habitat of origin for four species. Laboratory-reared specimens of *A.* cf. *citrinellus* and *A. centrarchus* were originally from the great lake, *A. siquia* and *H. nematopus* from the crater lake. Along the main axis of variation (PC1, explaining approximately 80% of the total variance), specimens cluster by habitat of origin, independent of rearing condition. PC1 scores were significantly affected by species identity (two-way ANOVA, *P* < 0.001) but also by rearing condition (wild-caught vs. laboratory-reared, *P* = 0.031) and their interaction (*P* = 0.027). Based on the sums of squares from the ANOVA table, we calculated the proportions of variance explained by either species identity or rearing condition. Overall, 85% of the total variance was explained by species identity, suggesting that cone opsin expression patterns are largely genetically determined. Note that the same test using PC2 scores produced very similar results with 64% of the total variance explained by species identity. However, there is also evidence that levels of phenotypic plasticity vary among species since the interaction term was significant. This can be observed when comparing *A. siquia*, where wild-caught and laboratory-reared specimens segregate along PC2 with *H. nematopus*, which shows almost no variation. Note that for laboratory-reared *A.* cf. *citrinellus*, qPCR data is shown that was obtained from Torres-Dowdall et al. (2017).

Supplementary Table 1: Morphological and ecological features of all study species. Within a food web, the position of organisms is defined by the trophic level based on their feeding behavior. A value of 2 is characteristics for herbivores, values of 3 and 4 are characteristic of primary and secondary carnivores. Data was obtained from www.fishbase.org.

| Species | Max length [cm] | Habitat | Trophic level | Polychromatism |
| --- | --- | --- | --- | --- |
| *A.* cf. *citrinellus* | 24,4 | Benthopelagic | 3,2 ± 0,47 | Dark & Gold morphs |
| *A. centrarchus* | 11,0 | Benthopelagic | 2,6 ± 0,28 | - |
| *A. siquia* | 7,9 | Benthopelagic | 2,3 ± 0,2 | Sexual dimorphism |
| *P. managuensis* | 55,0 | Benthopelagic | 4,0 ± 0,59 | - |
| *H. nematopus* | 14,0 | Demersal | 2,0 ± 0,00 | - |
| *H. nicaraguensis* | 16,5 | Benthopelagic | 2,7 ± 0,34 | Sexual dimorphism |
| *A. rostratus* | 18,5 | Benthopelagic | 2,6 ± 0,27 | - |

Supplementary Table 2: Sampling locations and sample sizes for all species.

|  | Crater lake Xiloá | Lake Nicaragua | Lake Managua | San Juan River | Punta Gorda River |
| --- | --- | --- | --- | --- | --- |
| GPS coordinates | N 12°12.502' | N 11°55.090' | N 12°13.197' | N 10°56.556' | N 11°29.538' |
|  | W 86°18.573' | W 85°55.012' | W 86°17.313' | W 83°43.599' | W 84°28.405' |
| *A.* cf. *citrinellus* | 5 |  | 4 | 6 |  |
| *A. centrarchus* | 6 | 3 |  | 6 |  |
| *A. siquia* | 6 | 6 |  | 6 | 6 |
| *P. managuensis* | 5 | 6 |  | 6 |  |
| *H. nematopus* | 6 | 6 |  |  | 5 |
| *H. nicaraguensis* | 6 |  | 6 | 6 |  |
| *A. rostratus* | 6 | 5 |  | 6 |  |

Supplementary Table 3 (provide in a separate Excel sheet): Total number of raw reads, reads mapped to the Midas cichlid reference genome and reads mapped to each cone opsin and cyp27c1.

Supplementary Table 4: Variable sites within species leading to amino acid substitutions in RH2Aβ and LWS opsin proteins. In SWS2A, non-synonymous substitutions were not found in any species. Grey boxes indicate sites under positive selection based on random site models of codon evolution in PAML. Numbering of amino acid sites is based on bovine RH1.

|  | protein | RH2Aβ | | |  | LWS | | | | | | | |
| --- | --- | --- | --- | --- | --- | --- | --- | --- | --- | --- | --- | --- | --- |
|  | nucleotide position | 559 | 664 | 767 |  | 77 | 157 | 172 | 280 | 656 | 680 | 688 | 898 |
| Species | n |  |  |  |  |  |  |  |  |  |  |  |  |
| *A.* cf. *citrinellus* | 15 |  |  |  |  |  |  |  |  |  |  |  |  |
| *A. centrarchus* | 15 |  |  | a/g |  |  |  |  |  |  |  | a/g |  |
| *A. siquia* | 24 |  |  |  |  |  | t/g | c/t |  |  |  |  |  |
| *P. managuensis* | 14 |  |  |  |  |  |  |  |  |  |  |  |  |
| *H. nematopus* | 17 |  | t/g |  |  | c/t | t/g | c/t |  | c/t |  |  | a/g |
| *H. nicaraguensis* | 18 |  |  |  |  |  |  |  | a/g |  | c/t |  |  |
| *A. rostratus* | 17 | a/c |  |  |  |  |  |  |  |  |  |  |  |
| amino acid substitution | | I179L | V214F | R248K |  | A10V | S40A | F45L | V81I | V206A | I214T | G217S | A287T |
| location | |  | TM5 | TM6 |  |  | TM1 | TM1 | TM2 | TM5 | TM5 | TM5 | TM7 |
|  |  |  |  |  |  |  |  |  |  |  |  |  |  |

Supplementary Table 5: Variable SWS2A residues across species. Numbering of amino acid sites is based on bovine RH1.

|  | protein | SWS2A | | | | | | | | | |
| --- | --- | --- | --- | --- | --- | --- | --- | --- | --- | --- | --- |
|  | amino acid residue | 13 | 42 | 47 | 50 | 101 | 131 | 155 | 220 | 232 | 248 |
| Species | n |  |  |  |  |  |  |  |  |  |  |
| *A.* cf. *citrinellus* | 15 | V | A | V | F | R | I | A | I | M | K |
| *A. centrarchus* | 15 | V | A | V | F | R | I | A | I | M | K |
| *A. siquia* | 24 | V | A | V | F | R | I | A | L | M | K |
| *P. managuensis* | 17 | V | A | V | F | R | I | A | I | M | K |
| *H. nematopus* | 17 | V | V | V | F | R | V | A | L | M | K |
| *H. nicaraguensis* | 18 | V | V | I | F | R | V | A | L | M | K |
| *A. rostratus* | 17 | I | A | I | C | K | I | V | I | L | R |
|  | location |  | TM1 | TM1 | TM1 | E-1 | TM3 | TM4 | TM5 | C-3 | TM6 |

Supplementary Table 6: Variable RH2Aβ residues across species. Numbering of amino acid sites is based on bovine RH1.

|  | protein | RH2Aβ | | | | | | |
| --- | --- | --- | --- | --- | --- | --- | --- | --- |
|  | amino acid residue | 39 | 104 | 151 | 179 | 214 | 228 | 248 |
| Species | n |  |  |  |  |  |  |  |
| *A.* cf. *citrinellus* | 15 | L | I | T | L | V | M | R |
| *A. centrarchus* | 15 | L | I | T | I | V | M | R/K |
| *A. siquia* | 24 | L | I | T | L | V | M | K |
| *P. managuensis* | 17 | L | I | T | L | V | L | R |
| *H. nematopus* | 17 | F | V | S | I | V/F | M | K |
| *H. nicaraguensis* | 18 | F | V | S | I | V | M | K |
| *A. rostratus* | 17 | L | I | T | I/L | V | M | K |
|  | location | TM1 | E-1 | TM4 | E-2 | TM5 | C-3 | TM6 |

Supplementary Table 7: Variable LWS residues across species. Numbering of amino acid sites is based on bovine RH1.

|  | protein | LWS | | | | | | | | | | | | | | | | | |
| --- | --- | --- | --- | --- | --- | --- | --- | --- | --- | --- | --- | --- | --- | --- | --- | --- | --- | --- | --- |
|  | amino acid residue | 40 | 45 | 52 | 63 | 88 | 92 | 111 | 115 | 155 | 162 | 164 | 166 | 169 | 213 | 214 | 217 | 218 | 282 |
| Species | n |  |  |  |  |  |  |  |  |  |  |  |  |  |  |  |  |  |  |
| *A.* cf. *citrinellus* | 15 | S | L | T | M | L | S | I | Y | A | V | A | F | S | I | I | S | I | A |
| *A. centrarchus* | 15 | S | L | T | A | L | T | V | F | S | V | S | F | A | I | I | G/S | V | A |
| *A. siquia* | 24 | S/A | F/L | T | M | L | S | I | Y | A | V | S | F | A | I | I | G | V | A |
| *P. managuensis* | 17 | A | F | V | A | V | S | I | Y | A | V | S | F | A | I | I | G/A | I | A |
| *H. nematopus* | 17 | S/A | F/L | T | M | I | S | I | Y | S | A | A | F | A | I | I | S | I | S |
| *H. nicaraguensis* | 18 | S | L | T | M | L | S | I | Y | A | A | A | F | A | I | I/T | G | I | S |
| *A. rostratus* | 17 | S | L | V | A | I | T | V | Y | G | V | S | V | A | F | I | G | V | A |
|  | location | TM1 | TM1 | TM1 | TM1 | TM2 | TM2 | TM3 | TM3 | TM4 | TM4 | TM4 | TM4 | TM4 | TM5 | TM5 | TM5 | TM5 | E-3 |

Supplementary Table 8: LRT of positive selection (random sites model in PAML) for three cone opsin coding sequences.

|  |  |  |  |  | Loglikelihood ratio tests | | Parameters under M2 | |  |
| --- | --- | --- | --- | --- | --- | --- | --- | --- | --- |
| Gene | no | le | M0 tree length | ω_M0_ | M3/M0 | M2/M1 | ω_0_(p_0_) | ω_2_(p_2_) | Positively selected sites (M2, BEB)^a^ |
| sws2a | 137 | 1,053 | 0.118 | 0.212 | 1.474^NS^ | 0.0001^NS^ | 0.003(0.79) | 1(0.21) |  |
| rh2aβ | 138 | 1,059 | 0.131 | 0.101 | 10.989* | 3.004^NS^ | 0.064(0.996) | 9.857(0.004) | 179 |
| lws | 156 | 1,071 | 0.199 | 0.534 | 85.142*** | 43.665*** | 0.133(0.963) | 12.773(0.037) | **40**,**45**,**52**,63,**88**,**155**,**164^b^**,**217**,218 |

no: number of sequences, le: length of sequences.

**P < 0.05; ***P < 0.001; ^NS^P > 0.05*.

^a^Only sites with a posterior probability higher than 80% are reported. If the posterior probability of a site belonging to the positively selected class is higher than 0.9, it is shown in bold. Numbering of amino acid sites is based on bovine RH1.

^b^Sites directed into the chromophore binding pocket.

***Supplementary References***

Härer A., Torres-Dowdall J., Meyer A. (2017) Rapid adaptation to a novel light environment: The importance of ontogeny and phenotypic plasticity in shaping the visual system of Nicaraguan Midas cichlid fish (Amphilophus citrinellus spp.). Molecular Ecology 26, 5582-5593.

Torres-Dowdall J., Pierotti M.E.R., Härer A. *et al.* (2017) Rapid and parallel adaptive evolution of the visual system of Neotropical Midas cichlid fishes. Molecular Biology and Evolution, doi: 10.1093/molbev/msx1143.
